# The comparative burden of mild, moderate and severe Fibromyalgia: results from a cross-sectional survey in the United States

**DOI:** 10.1186/1477-7525-9-71

**Published:** 2011-08-22

**Authors:** Caroline Schaefer, Arthi Chandran, Meghan Hufstader, Rebecca Baik, Michael McNett, Don Goldenberg, Robert Gerwin, Gergana Zlateva

**Affiliations:** 1 Health Economics and Outcomes Research, Covance Market Access Services Inc., Gaithersburg, MD USA; 2 Primary Care Health Economics and Outcomes Research, Pfizer Global Health Economics, New York, NY USA; 3 APAC Centers for Pain Management, Chicago, IL USA; 4 Division of Rheumatology, Newton-Wellesley Hospital, Newton, MA USA; Division of Rheumatology, Tufts Medical Center, Tufts University School of Medicine, Boston, MA USA; 5 Pain & Rehabilitation Medicine, Bethesda, MD USA

**Keywords:** Fibromyalgia, Quality of Life, Patient Outcomes Assessment, Burden of Illness, Health Resources

## Abstract

**Background:**

Fibromyalgia (FM) is characterized by chronic, widespread pain, fatigue, and other symptoms; yet few studies have comprehensively assessed its humanistic burden. This observational study evaluates the impact of FM severity on patients' symptoms, health-related quality of life (HRQoL), and productivity in the United States.

**Methods:**

203 FM subjects were recruited from 20 physician offices. Subjects completed a questionnaire including the EuroQol 5D (EQ-5D), Fibromyalgia Impact Questionnaire (FIQ), Multidimensional Assessment of Fatigue (MAF), Medical Outcomes Study Sleep Scale (MOS-SS), and Hospital Anxiety and Depression Scale (HADS) and questions about demographics, pain and other symptoms, HRQoL and productivity. FIQ total scores were used to define FM severity, with 0- < 39, 39- < 59, and 59-100, representing mild, moderate, and severe FM, respectively. Sites recorded subjects' clinical characteristics and FM treatment on case report forms using medical records. Summary statistics were calculated for continuous variables and frequency distributions for categorical variables. Differences across FM severity groups were evaluated using the Kruskal-Wallis or Chi-square tests. Statistical significance was evaluated at the 0.05 level.

**Results:**

Mean (SD) age was 47.9 (10.9); 95% were female. Most (92%) were prescribed medication for FM; 24% and 66% reported moderate and severe FM, respectively. Mean (SD) scores were: 6.3 (2.1) for pain intensity; 0.35 (0.35) for EQ-5D; 30.7 (14.2) for MAF; 57.5 (18.4) for MOS-SS Sleep Problems Index; 10.2 (4.8) for HADS anxiety and 9.4 (4.4) for HADS depression. Subjects with worse FM severity reported significantly increased pain severity, HRQoL, fatigue, sleep disturbance, anxiety and depression (p < 0.001). Overall, 50% of subjects reported some disruption in their employment due to FM; this differed across severity levels (p < 0.001). Employed subjects missed a mean (SD) of 1.8 (3.9) workdays during the past 4 weeks; this also differed across severity levels (p = 0.03).

**Conclusions:**

FM imposes a substantial humanistic burden on patients in the United States, and leads to substantial productivity loss, despite treatment. This burden is higher among subjects with worse FM severity.

## Background

Fibromyalgia (FM) is characterized by chronic, widespread, musculoskeletal pain and is estimated to affect 2 to 6 percent of the general population in the United States (US), primarily women [1,2]. FM patients often also experience a number of other symptoms, such as fatigue, sleep disturbance, headache, irritable bowel syndrome, cognitive dysfunction, and mood disorders [1-4]. Consequently, FM patients frequently report limitations in physical function, and activities of daily living (ADL), as well as decrements in other physical and mental domains of health-related quality of life (HRQoL) [5-7]. In addition, recent studies also established an association between pain in FM and mental symptoms that could not be found in patients with neuropathic pain [4,8]. Another study found pain intensity reduction to be associated with improvements in other FM outcomes [9]. Lastly, a negative impact of FM on work productivity also has been reported in the literature [5,7,10,11].

The Outcome Measures in Rheumatology Clinical Trials (OMERACT) workgroup on FM has identified domains that should be captured in clinical trials for FM therapies and aspects of domains and outcome measures that should be part of a concerted research agenda for FM researchers [12]. The identified domains included pain, patient global health, fatigue, HRQoL, multidimensional function, sleep, depression, physical function, tenderness, dyscognition, and anxiety.

Recent studies in Europe have explored the impact of FM on HRQoL and other OMERACT domains more comprehensively; [13] however, few cross-sectional studies have been conducted that include a comprehensive assessment of the burden of illness associated with FM in the US. Previous US studies have examined the impact of FM on patients' HRQoL [14] or focused on one aspect of disease burden, such as limitations in functioning [3] or sleep problems [15,16]. In particular, there is a gap in the literature that examines the disease burden by FM severity levels.

The objective of this study was to better understand FM patients in the US by conducting a comprehensive evaluation of their clinical and comorbid profile, and the impact of FM severity on HRQoL, symptom domains (such as, pain, sleep, depression), and productivity loss for patients and caregivers.

## Methods

### Study Design

This cross-sectional, observational study included 203 FM subjects recruited from 20 community-based physician offices (8 primary care physicians, 6 rheumatologists, 3 neurologists, and 3 psychiatrists) in the US. Subjects were required to have the following: a prior FM diagnosis by a rheumatologist or pain specialist, experienced widespread pain (above and below the waist and on both sides of the body) for 3 months or more, experienced pain in the past 24 hours, and been in treatment at the enrolling physician's practice for at least 3 months. Additionally, all subjects had to be between 18 and 65 years of age and were excluded if they had participated in an investigational drug study within 30 days prior to the survey or had a concomitant illness unrelated to FM that was likely to confound the assessment of FM, such as osteoarthritis, lupus, or rheumatoid arthritis.

The protocol was approved by a central institutional review board and all subjects provided written informed consent. No medical interventions were required by the study protocol. Site study staff identified potential subjects during routine visits. Once consent was obtained, subjects were asked to complete a questionnaire that assessed FM's impact on the core OMERACT domains, as well as productivity (subject questionnaire). Site staff completed a case report form (CRF) based on review of the subject's medical records and assessed FM using the Manual Tender Point Survey (MTPS) exam [17]. Data captured on the CRF included subject's demographics, clinical characteristics, current and previous medications for FM, and frequency of the following: FM-related office visits, diagnostic tests, emergency room visits, and hospitalizations. The subject questionnaire and CRF were labeled with a study-specific subject identifier that was assigned at enrollment to allow linking of the subjects' clinical and HRQoL data for analysis.

#### Subject Questionnaire

The subject questionnaire included 5 validated HRQoL questionnaires: the EuroQol (EQ-5D) [18], the Fibromyalgia Impact Questionnaire (FIQ) [19,20], the Multidimensional Assessment of Fatigue (MAF) [21], the Medical Outcomes Study Sleep Scale (MOS-SS) [22], and the Hospital Anxiety and Depression Scale (HADS) [23].

The EQ-5D describes HRQoL across 5 dimensions: mobility, self-care, performance of usual activities, pain or discomfort, and anxiety or depression [18]. Subjects indicate their health state by selecting the most appropriate response (i.e., no problems, some problems, or extreme problems) for each statement within each dimension. Health state valuation scores range from -0.594 to 1.00 with a higher score indicating better HRQoL [18].

The FIQ measures FM subject's status, progress, and outcomes in 10 areas: physical impairment, feeling good, work missed, doing work, pain, fatigue/tired, rested, stiffness, anxiety, and depression [19]. The impact of FM on activities of daily living (ADLs) (i.e., preparing meals, doing laundry, climbing stairs, shopping, yard work, driving a car, visiting friends, washing dishes, vacuuming, making beds, and walking several blocks) was evaluated using the FIQ Physical Impairment Score which ranges from 0 to 10. The FIQ total score reflects all ten areas and ranges from 0 to 100. Higher scale scores indicate a greater impact of the disease.

The MAF measures 4 dimensions of fatigue: severity, distress, timing, and degree of interference in ADLs [21]. The scores from the 4 dimensions are summed to calculate the Global Fatigue Index (GFI), which ranges from 1 (indicating no fatigue) to 50 (indicating severe fatigue).

The MOS-SS includes the 9-item Sleep Problems Index score and 6 subscales: sleep disturbances, snoring, awakening short of breath or with a headache, sleep adequacy, somnolence, and sleep quantity [22]. With the exception of sleep quantity, the subscales and the 9-item Sleep Problems Index scores range from 0 to 100. The sleep quantity scale is the mean number of hours of sleep per night reported over the past week. Higher scores represent more of the concept being measured.

The HADS is designed to detect the presence and severity of mood disorders and has been used extensively in a variety of populations [23]. The HADS Anxiety and Depression subscale scores range from 0 to 21, with higher scores representing more symptoms and poorer emotional well-being. Scores of 0 to 7 on either subscale are considered normal, 8 to 10 are considered mild, 11 to 14 are considered moderate, and 15 to 21 are considered severe.

To assess pain intensity, subjects were asked to rate their average pain due to FM over the past 24 hours with an 11-point numeric rating scale ranging from 0 (indicating no pain) to 10 (indicating pain as bad as you can imagine). Higher scores indicate greater pain severity. Based on previous analyses, scores of 0 to 3 are considered mild, 4 to 6 are considered moderate, and 7 to 10 are considered severe [24].

Study-specific questions also were developed to assess the following: average pain over the past 24 hours, impact of FM on daily life, overall health status, cognitive function, and subject and caregiver productivity over the past four weeks (e.g., subject-reported time missed from work, reduced productivity at work, hours of unpaid help from caregiver).

#### Statistical Analysis

Means, standard deviations (SD), medians, and ranges were calculated for continuous variables and frequency counts and percentages were calculated for categorical variables. To evaluate the impact of FM severity, outcomes reported on the subject questionnaires and CRFs were compared across 3 FM severity level classifications (i.e., mild, moderate, and severe) using the Kruskal-Wallis test. Categorical outcomes were compared across FM severity level using a Chi-square test. FM severity level was defined based on the subject's FIQ total score where 0 to < 39 was classified as mild, 39 to < 59 was classified as moderate, and 59 to 100 was classified as severe [20]. Statistical significance was evaluated at the 0.05 level. The data were held and analyzed by Covance Inc. All analyses were performed using SAS version 9.1 [25].

## Results

### Demographic Characteristics

The study enrolled 203 subjects. Subject demographics are summarized in Table 1. The mean age was 47.9, and almost all (95%) were female. Nearly half (41%) of the subjects were employed either full-time or part-time for pay and nearly another half (41%) were disabled, unemployed, or retired. Age and gender did not differ significantly across FM severity levels. Employment status differed across severity levels (p < 0.001) with a higher proportion of mild FM subjects (71.4%) being employed for pay either full-time or part-time than moderate (61.2%) or severe (28.5%) subjects. Additionally, a higher proportion of severe FM subjects (52.6%) reported being disabled, unemployed, or retired than mild (9.6%) or moderate (22.4%).

**Table 1:** Demographic Characteristics, Overall and by Fibromyalgia Severity Level

		Severity Level			
Characteristic	Overall (n = 203)	Mild (n = 21)	Moderate (n = 49)	Severe (n = 133)	p-value^a^
Age, years					0.148
mean (SD)	47.9 (10.9)	48.6 (11.9)	45.3 (11.0)	48.7 (10.6)	
median	50.0	53.0	46.0	50.0	
range	19.0 - 65.0	28.0 - 65.0	19.0 - 64.0	21.0 - 65.0	
Gender, n (%)					0.621
Male	11 (5.4)	2 (9.5)	3 (6.1)	6 (4.5)	
Female	192 (94.6)	19 (90.5)	46 (93.9)	127 (95.5)	
Employment status, n (%)					< 0.001
Employed, full-time	57 (28.1)	13 (61.9)	18 (36.7)	26 (19.5)	
Employed, part-time	26 (12.8)	2 (9.5)	12 (24.5)	12 (9.0)	
Disabled	55 (27.1)	0 (0.0)	7 (14.3)	48 (36.1)	
Full-time homemaker	25 (12.3)	4 (19.0)	5 (10.2)	16 (12.0)	
Unemployed	16 (7.9)	1 (4.8)	1 (2.0)	14 (10.5)	
Retired	12 (5.9)	1 (4.8)	3 (6.1)	8 (6.0)	
Other	8 (3.9)	0 (0.0)	2 (4.1)	6 (4.5)	
Student	4 (2.0)	0 (0.0)	1 (2.0)	3 (2.3)	

^a ^Chi-square test or the Kruskal-Wallis test, as appropriate.

Source: Subject Questionnaire.

### Clinical Characteristics

On average, subjects reported having FM symptoms for longer (mean of 10.7 years) than having an FM diagnosis (mean of 6.9 years) or than having received prescription medications for FM (mean of 6.3 years) (Table 2).

**Table 2:** Clinical Characteristics and Treatment Patterns, Overall and by Fibromyalgia Severity Level

		Severity Level			
Characteristic	Overall (n = 203)	Mild (n = 21)	Moderate (n = 49)	Severe (n = 133)	p-value^a^
Time since first experienced FM symptoms, years					0.684
mean (SD)	10.7 (8.1)	10.8 (6.3)	10.1 (8.1)	10.9 (8.3)	
median (range)	9.0 (0.0 - 48.0)	10.0 (3.0 - 23.0)	9.0 (1.0 - 32.0)	9.0 (0.0 - 48.0)	
Time since diagnosis, years					
mean (SD)	6.9 (6.5)	7.4 (6.1)	6.2 (6.2)	7.0 (6.6)	0.604
median (range)	6.0 (0.0 - 48.0)	6.0 (0.0 - 22.0)	4.0 (0.0 - 30.0)	6.0 (0.0 - 48.0)	
Time since first prescription for FM, years					0.670
mean (SD)	6.3 (6.2)	7.2 (6.6)	5.9 (6.6)	6.2 (6.0)	
median (range)	4.5 (0.0 - 30.0)	5.0 (0.0 - 22.0)	3.0 (0.0 - 30.0)	5.0 (0.0 - 21.0)	
Number of positive MTPS points					0.036
mean (SD)	14.7 (3.4)	13.7 (4.1)	13.8 (3.8)	15.2 (3.0)	
median (range)	16.0 (4.0 - 18.0)	16.0 (5.0 - 18.0)	15.0 (4.0 -18.0)	16.0 (4.0 - 18.0)	
Number of comorbid conditions^b^					0.112
mean (SD)	4.2 (2.4)	2.9 (1.6)	4.1 (2.3)	4.4 (2.6)	
median (range)	4.0 (1.0-10.0)	3.0 (1.0-7.0)	4 (1.0-10.0)	4.0 (1.0-10.0)	
Average pain intensity, n (%)					< 0.001
Mild (0-3)	20 (9.9)	12 (57.1)	7 (14.3)	1 (0.8)	
Moderate (4-6)	74 (36.6)	6 (28.6)	24 (49.0)	44 (33.3)	
Severe (7-10)	108 (53.5)	3 (14.3)	18 (36.7)	87 (65.9)	
Number of physician visits (per year)					< 0.001
mean (SD)	16.9 (17.9)	9.7 (11.3)	11.6 (11.2)	19.9 (19.9)	
median (range)	12.0 (4.0 - 132.0)	4.0 (4.0 - 48.0)	8.0 (4.0 - 52.0)	16.0 (4.0 - 132.0)	
Number of medications over the past 3 months, n (%)					
≥ 1 medication	37 (91.6)	19 (90.5)	43 (87.8)	124 (93.2)	0.487
≥ 3 medications	48 (47.3)	3 (14.3)	22 (44.9)	71 (53.4)	0.004

^a ^Chi-square test or the Kruskal-Wallis test, as appropriate

^b ^Among subjects with at least one comorbid condition (n = 190 overall; n = 15 mild; n = 47 moderate; n = 128 severe)

Source: Clinical CRF.

Severe FM subjects, on average, had more positive MTPS points with mean of 15.2 compared to mild subjects with a mean of 13.7 and moderate subjects with a mean of 13.8 (p = 0.036). Additionally, over half (54%) of the subjects reported severe average pain intensity with a higher proportion of severe FM subjects (66%) reporting severe average pain intensity compared to mild (14%) and moderate (37%) FM subjects (p < 0.001) (Table 2).

Subjects were actively seeking care at the time of the study. Most (92%) were taking 1 or more prescription medications for FM and approximately half (47%) were taking 3 or more. In addition, subjects reported a mean of 16.9 physician visits over the past year (Table 2).

### Comorbid Conditions

Overall, subjects had a mean number of 4.2 comorbid conditions. While not significant (p = 0.112), there was a trend toward an increasing number of comorbid conditions as FM severity worsened from 2.9 for mild FM subjects to 4.4 for severe FM subjects (Table 2). Approximately half or more subjects had comorbid conditions of sleep disturbance/insomnia (68%), depressive symptoms (58%), headache/migraine (52%), or anxiety (50%) as reported by the physicians on the CRF (Table 3). Depressive symptoms (p < 0.001), anxiety (p = 0.002), chronic fatigue syndrome (p = 0.003), and major depressive disorder (p = 0.024) differed significantly across FM severity levels. For these conditions, the proportion of subjects with the condition increased as FM severity increased, except for chronic fatigue syndrome, which was most frequently reported among subjects with moderate FM.

**Table 3:** Comorbid Conditions, Overall and by Fibromyalgia Severity Level

		Severity Level			
Comorbid condition, n (%)^a^	Total (n = 203)	Mild (n = 21)	Moderate (n = 49)	Severe (n = 133)	p-value^b^
Sleep disturbance/Insomnia	138 (68.0)	11 (52.4)	35 (71.4)	92 (69.2)	0.259
Depressive Symptoms	117 (57.6)	5 (23.8)	20 (40.8)	92 (69.2)	< 0.001
Headache/migraine	105 (51.7)	10 (47.6)	26 (53.1)	69 (51.9)	0.915
Anxiety	101 (49.8)	4 (19.0)	20 (40.8)	77 (57.9)	0.002
Chronic Fatigue Syndrome	86 (42.4)	2 (9.5)	26 (53.1)	58 (43.6)	0.003
Irritable Bowel Syndrome	74 (36.5)	5 (23.8)	17 (34.7)	52 (39.1)	0.384
Restless Leg Syndrome	58 (28.6)	3 (14.3)	16 (32.7)	39 (29.3)	0.281
Cognitive Dysfunction	55 (27.1)	2 (9.5)	15 (30.6)	38 (28.6)	0.154
Major Depressive Disorder	47 (23.2)	0 (0.0)	11 (22.4)	36 (27.1)	0.024
Raynaud's Syndrome	15 (7.4)	2 (9.5)	5 (10.2)	8 (6.0)	0.584

^a ^Subjects may have more than one condition; thus, percents may sum to more than 100%.

^b ^Chi-square test.

Source: Clinical CRF.

### Impact of FM on Health-Related Quality of Life and Core FM Symptoms

When asked to rank areas of daily life affected by FM, most subjects reported pain (91%) and lack of energy/fatigue (87%). Over half reported that FM impacted their sleep (54%) and caused them difficulty walking, moving, or exercising (51%) (Table 4).

**Table 4:** Impact of Fibromyalgia on Areas of Daily Life, Overall and by Fibromyalgia Severity Level

Areas of Daily Life Impacted by FM n (%)		Severity Level			
	Total (n = 203)	Mild (n = 21)	Moderate (n = 49)	Severe (n = 133)	p-value^a^
Pain	183 (90.6)	19 (90.5)	43 (89.6)	121 (91.0)	0.960
Lack of energy/fatigue	175 (86.6)	5 (23.8)	23 (47.9)	75 (56.4)	0.019
Sleep disturbance	109 (54.0)	17 (81.0)	44 (91.7)	114 (85.7)	0.421
Difficulty moving, walking, or exercising	103 (51.0)	13 (61.9)	24 (50.0)	72 (54.1)	0.658
Limited daily life and household activities	92 (45.5)	9 (42.9)	14 (29.2)	32 (24.1)	0.187
Stiffness	85 (42.1)	13 (61.9)	31 (64.6)	41 (30.8)	< 0.001
Depression	81 (40.1)	12 (57.1)	20 (41.7)	45 (33.8)	0.105
Tender at touch	77 (38.1)	4 (19.0)	16 (33.3)	61 (45.9)	0.036
Problems with attention	55 (27.2)	2 (9.5)	3 (6.3)	29 (21.8)	0.030
Anxiety	34 (16.8)	6 (28.6)	22 (45.8)	64 (48.1)	0.247
Other	9 (4.5)	1 (4.8)	0 (0.0)	8 (6.0)	0.223

^a ^Chi-square test.

Source: Subject Questionnaire.

The overall mean average pain intensity over the past 24 hours was 6.3. Average Pain Intensity differed across FM severity levels (p < 0.001) with mean scores of 3.4 for mild, 5.6 for moderate, and 7.0 for severe (Table 5).

**Table 5:** Health-Related Quality of Life Scores, Overall and by Fibromyalgia Severity Level

		Severity Level			
Scale	Overall (N = 203)	Mild (N = 21)	Moderate (N = 49)	Severe (N = 133)	p-value^a^
**EQ-5D**					
Health State Valuation					< 0.001
n	203	21	49	133	
mean (SD)	0.35 (0.35)	0.76 (0.11)	0.57 (0.21)	0.20 (0.31)	
median (range)	0.52 (0.2- 1.0)	0.76 (0.6 - 1.0)	0.62 (0.0 - 1.0)	0.09 (0.2 - 0.8)	
**Overall Health Status** **Ratings**					
Current Overall Health					< 0.001
n	203	21	49	133	
mean (SD)	54.9 (21.0)	71.0 (21.5)	62.7 (16.9)	49.4 (20.1)	
median (range)	50.0 (2.0- 100.0)	75.0 (10.0 - 95.0)	65.0 (10.0 - 90.0)	50.0 (2.0 - 100.0)	
Pain-Free Overall Health					0.001
n	202	21	49	132	
mean (SD)	79.3 (20.4)	84.6 (23.9)	84.2 (19.4)	76.6 (19.8)	
median (range)	89.5 (0.0-100.0)	90.0 (10.0 - 100.0)	90.0 (0.0 - 100.0)	80.0 (15.0 - 100.0)	
**Average Pain Intensity**					< 0.001
n	202	21	49	132	
mean (SD)	6.3 (2.1)	3.4 (2.0)	5.6 (1.8)	7.0 (1.6)	
median (range)	7.0 (1.0-100.0)	3.0 (1.0 - 7.0)	6.0 (2.0 - 8.0)	7.0 (3.0 - 10.0)	
**FIQ**					n/a^b^
Total Score					
n	203	21	49	133	
mean (SD)	63.2 (19.0)	23.9 (9.5)	49.3 (5.1)	74.5 (9.1)	
median (range)	67.4 (6.0-99.0)	25.9 (6.0 - 38.1)	48.6 (39.9 - 58.9)	73.4 (59.1 - 99.0)	
Physical Impairment					n/a^b^
n	203	21	49	133	
mean (SD)	5.1 (2.4)	2.4 (2.6)	3.8 (2.1)	6.0 (1.8)	
median (range)	5.0 (0.0-10.0)	1.0 (0.0 - 10.0)	4.0 (0.0 - 10.0)	6.0 (0.0 - 10.0)	
Feel Good					n/a^b^
n	203	21	49	133	
mean (SD)	7.1 (2.8)	3.1 (2.9)	6.0 (2.3)	8.2 (2.1)	
median (range)	7.1 (0.0-10.0)	2.9 (0.0 - 10.0)	5.7 (0.0 - 10.0)	8.6 (0.0 - 10.0)	
Work Missed					n/a^b^
n	202	21	49	132	
mean (SD)	4.5 (3.5)	0.9 (2.3)	2.2 (2.3)	5.9 (3.2)	
median (range)	4.3 (0.0-10.0)	0.0 (0.0 - 10.0)	1.4 (0.0 - 10.0)	6.4 (0.0 - 10.0)	
Do Work					n/a^b^
n	201	21	49	131	
mean (SD)	6.4 (2.7)	1.8 (1.6)	4.7 (1.8)	7.8 (1.6)	
median (range)	7.0 (0.0-10.0)	2.0 (0.0 - 5.0)	5.0 (0.0 - 7.0)	8.0 (4.0 - 10.0)	
Pain					n/a^b^
n	203	21	49	133	
mean (SD)	7.0 (2.3)	3.0 (1.6)	6.0 (1.8)	7.9 (1.6)	
median (range)	7.0 (1.0-10.0)	3.0 (1.0 - 7.0)	6.0 (2.0 - 9.0)	8.0 (3.0 - 10.0)	
Fatigue/Tired					n/a^b^
n	203	21	49	133	
mean (SD)	7.9 (2.3)	3.5 (2.0)	6.8 (1.9)	8.9 (1.2)	
median (range)	8.0 (0.0-10.0)	4.0 (0.0 - 7.0)	7.0 (3.0 - 10.0)	9.0 (4.0 - 10.0)	
Rested					n/a^b^
n	203	21	49	133	
mean (SD)	7.9 (2.3)	4.1 (2.3)	7.1 (1.8)	8.8 (1.6)	
median (range)	8.0 (0.0-10.0)	5.0 (0.0 - 8.0)	7.0 (3.0 - 10.0)	9.0 (2.0 - 10.0)	
Stiffness					n/a^b^
n	203	21	49	133	
mean (SD)	7.2 (2.4)	3.6 (2.3)	6.4 (1.7)	8.1 (1.9)	
median (range)	8.0 (0.0-10.0)	3.0 (0.0 - 9.0)	6.0 (2.0 - 10.0)	9.0 (2.0 - 10.0)	
Anxiety					n/a^b^
n	203	21	49	133	
mean (SD)	5.1 (3.2)	1.1 (1.7)	3.2 (2.2)	6.4 (2.8)	
median (range)	5.0 (0.0-10.0)	0.0 (0.0 - 6.0)	3.0 (0.0 - 7.0)	7.0 (0.0 - 10.0)	
Depression					n/a^b^
n	203	21	49	133	
mean (SD)	5.1 (3.2)	0.6 (0.8)	3.2 (2.4)	6.5 (2.6)	
median (range)	6.0 (0.0-10.0)	0.0 (0.0 - 2.0)	3.0 (0.0 - 9.0)	7.0 (0.0 - 10.0)	
**MOS Sleep**					
Sleep Problems Index					< 0.001
n	203	21	49	133	
mean (SD)	57.5 (18.4)	34.4 (13.6)	50.7 (16.9)	63.7 (15.6)	
median (range)	58.3 (13.3-95.6)	33.9 (13.3 - 65.6)	47.8 (20.6 - 88.9)	65.0 (24.4 - 95.6)	
Sleep Disturbance					< 0.001
n	203	21	49	133	
mean (SD)	56.4 (25.3)	31.6 (22.5)	49.0 (22.0)	63.1 (23.8)	
median (range)	56.3 (5.0-100.0)	26.3 (5.0 - 78.8)	42.5 (10.0 - 100.0)	66.3 (5.0 - 100.0)	
Sleep Adequacy					< 0.001
n	203	21	49	133	
mean (SD)	27.9 (23.6)	46.2 (21.8)	34.9 (24.4)	22.5 (21.6)	
median (range)	30.0 (0.0-90.0)	50.0 (0.0 - 80.0)	40.0 (0.0 - 90.0)	20.0 (0.0 - 70.0)	
Sleep Somnolence					< 0.001
n	203	21	49	133	
mean (SD)	52.3 (26.0)	27.6 (17.4)	45.0 (24.0)	58.8 (24.9)	
median (range)	53.3 (0.0-100.0)	20.0 (6.7 - 80.0)	40.0 (0.0 - 100.0)	60.0 (0.0 - 100.0)	
Snoring					0.026
n	201	21	49	131	
mean (SD)	43.3 (36.0)	22.9 (26.3)	46.1 (35.2)	45.5 (36.8)	
median (range)	40.0 (0.0-100.0)	20.0 (0.0 - 100.0)	40.0 (0.0 - 100.0)	40.0 (0.0 - 100.0)	
Shortness of Breath or Headache					0.001
n	202	21	49	132	
mean (SD)	34.3 (30.8)	14.3 (20.1)	29.4 (30.3)	39.2 (30.9)	
median (range)	40.0 (0.0-100.0)	0.0 (0.0 - 60.0)	20.0 (0.0 - 100.0)	40.0 (0.0 - 100.0)	
Sleep Quantity					0.002
n	194	21	49	124	
mean (SD)	6.3 (1.9)	6.8 (1.1)	6.8 (2.0)	6.0 (1.9)	
median (range)	6.0 (1.0-12.0)	7.0 (4.0 - 9.0)	7.0 (1.0 - 11.0)	6.0 (2.0 - 12.0)	
**MAF**					
Global Fatigue Index					< 0.001
n	203	21	49	133	
mean (SD)	30.7 (14.2)	16.5 (7.1)	26.3 (12.6)	34.6 (13.8)	
median (range)	35.8 (2.5-50.0)	17.5 (2.5 - 27.2)	29.6 (7.5 - 49.5)	39.4 (7.5 - 50.0)	
**HADS**					
Anxiety					< 0.001
n	203	21	49	133	
mean (SD)	10.2 (4.8)	5.5 (3.6)	7.3 (4.2)	12.0 (4.1)	
median (range)	11.0 (0.0-12.0)	4.0 (1.0 - 12.0)	7.0 (0.0 - 16.0)	12.0 (2.0 - 21.0)	
Depression					< 0.001
n	203	21	49	133	
mean (SD)	9.4 (4.4)	3.2 (2.7)	7.2 (2.6)	11.3 (3.8)	
median (range)	9.0 (0.0-21.0)	2.0 (0.0 - 9.0)	7.0 (1.0 - 12.0)	11.0 (2.0 - 21.0)	

^a ^Kruskal-Wallis test.

^b ^P-value not noted due to the association between severity level and FIQ score.

* Source: Subject Questionnaire.

**Figure 1:**
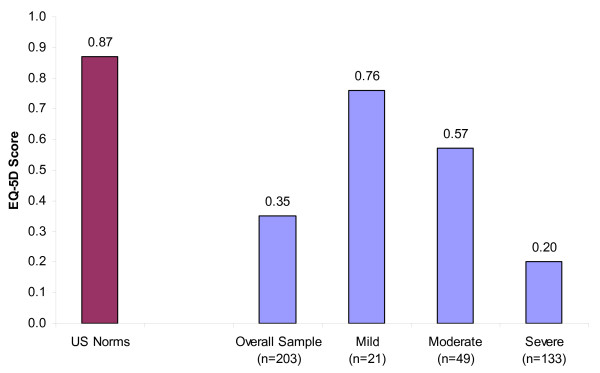
**Impact of Fibromyalgia on HRQoL: Mean Study Sample EQ-5D Scores Compared to US General Population**. Source: Subject Questionnaire and Fryback et al, 2007 (US Norm for age range 45-54) [28]. Note: Higher scores indicate better HRQoL.

With respect to overall health, subjects reported a mean EQ-5D score of 0.35 (Table 5; Figure 1). Subjects had significantly different EQ-5D scores across FM severity levels (p < 0.001; Table 5). Mild FM subjects had a mean EQ-5D score of 0.76, moderate subjects 0.57, and severe subjects 0.20.

The mean current overall health rating was 54.9, and subjects estimated that their overall health rating would be 24.4 points higher (Pain-Free Overall health mean of 79.3), on average, if they had complete relief of FM-related pain. The current Overall Health Score decreased as FM severity worsened (p < 0.001) as did subjects' estimates of their pain-free overall health (p < 0.001) (Table 5). Mild, moderate, and severe FM subjects estimated that their overall health rating would be 13.6, 21.5, and 27.2 points higher (Pain-Free Overall Health = 84.6, 84.2, and 76.6), respectively, if they had complete relief of FM-related pain.

The overall mean FIQ Total Score was 63.2 (Table 5), with 10 percent, 24 percent and 66 percent of subjects reporting mild, moderate, and severe scores, respectively. The highest mean FIQ subscale scores were observed in the following areas: rested 7.9, fatigue/tired 7.9, and stiffness 7.2 (Table 5). The mean Physical Impairment FIQ score was 5.1. Mild FM subjects reported a mean of 2.4, moderate subjects 3.8, and severe subjects 6.0, respectively.

**Figure 2:**
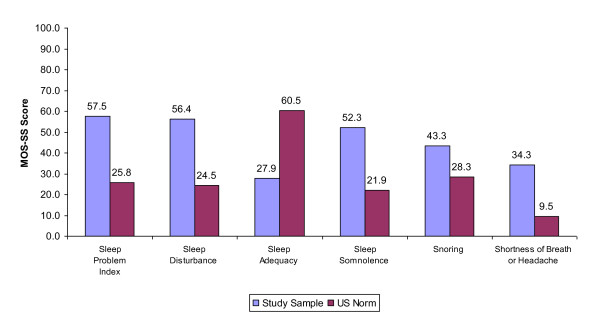
**Impact of Fibromyalgia on Sleep: Mean Study Sample MOS-SS Scores Compared to US General Population**. Source: Subject Questionnaire and Hays et al, 2005 (US Norms for age range 18-94) [26]. Note: Higher scores indicate more of the concept being measured. Higher scores represent worse outcomes on all scales except for sleep adequacy, where higher scores represent better outcomes.

Subjects reported a mean MOS-SS Sleep Problems Index score of 57.5 and Sleep Quantity of 6.3 hours (Table 5; Figure 2). The most affected area was Sleep Adequacy, with a mean score of 27.9. Subjects reported mean MOS Sleep Problems Index scores of 34.4, 50.7, and 63.7 for the respective FM severity groups, indicating increasing sleep problems with more severe FM (p < 0.001). Severe FM subjects also reported fewer hours of sleep (mean: 6.0 compared to mild 6.8 and moderate FM subjects 6.8; p = 0.002).

The mean HADS Anxiety score was 10.2 (Table 5), with 19 percent, 33 percent, and 19 percent of the subjects reporting mild, moderate and severe levels of anxiety, respectively. The mean HADS Depression score was 9.4, with 25 percent, 26 percent, and 13 percent of the subjects reporting mild, moderate, and severe levels of depression, respectively. Subjects indicated increased anxiety with more severe FM with mean HADS Anxiety scores of 5.5, 7.3, and 12.0 for mild, moderate, and severe FM, respectively (p < 0.001). Similarly the HADS Depression scores indicated increased depression with more severe FM with mean depression scores of 3.2, 7.2, and 11.3 for mild, moderate, and severe FM, respectively (p < 0.001).

Physicians reported cognitive dysfunction to be a comorbid condition for 27 percent of the study sample (Table 3) and 27 percent of subjects stated FM caused them to have problems with attention (Table 4). A majority of subjects reported that FM "moderately", "very much" or "totally" impacted their ability to remember (76%), concentrate (75%), think (64%), and make decisions (61%). Additionally, as severity level worsened, the impact of FM on these areas of cognitive function significantly increased (p < 0.001; Table 6).

**Table 6:** Subject-reported Impact of Fibromyalgia on Cognitive Function, Overall and by Fibromyalgia Severity Level

Cognitive Area	Overall (n = 203)	Mild (n = 21)	Moderate (n = 49)	Severe (n = 133)	p-value^a^
Concentrate, n (%)					< 0.001
Not at all	9 (4.4)	4 (19.0)	4 (8.2)	1 (0.8)	
A little	41 (20.2)	11 (52.4)	15 (30.6)	15 (11.3)	
Moderately	49 (24.1)	4 (19.0)	14 (28.6)	31 (23.3)	
Very much	82 (40.4)	2 (9.5)	15 (30.6)	65 (48.9)	
Totally	22 (10.8)	0 (0.0)	1 (2.0)	21 (15.8)	
Remember, n (%)					< 0.001
Not at all	11 (5.4)	5 (23.8)	4 (8.2)	2 (1.5)	
A little	38 (18.7)	10 (47.6)	14 (28.6)	14 (10.5)	
Moderately	43 (21.2)	4 (19.0)	9 (18.4)	30 (22.6)	
Very much	89 (43.8)	2 (9.5)	21 (42.9)	66 (49.6)	
Totally	22 (10.8)	0 (0.0)	1 (2.0)	21 (15.8)	
Make Decisions, n (%)					< 0.001
Not at all	24 (11.8)	9 (42.9)	4 (8.2)	11 (8.3)	
A little	56 (27.6)	9 (42.9)	24 (49.0)	23 (17.3)	
Moderately	51 (25.1)	2 (9.5)	13 (26.5)	36 (27.1)	
Very much	63 (31.0)	1 (4.8)	8 (16.3)	54 (40.6)	
Totally	9 (4.4)	0 (0.0)	0 (0.0)	9 (6.8)	
Think, n (%)					< 0.001
Not at all	19 (9.4)	8 (38.1)	7 (14.3)	4 (3.0)	
A little	54 (26.6)	9 (42.9)	16 (32.7)	29 (21.8)	
Moderately	53 (26.1)	3 (14.3)	14 (28.6)	36 (27.1)	
Very much	64 (31.5)	1 (4.8)	12 (24.5)	51 (38.3)	
Totally	13 (6.4)	0 (0.0)	0 (0.0)	13 (9.8)	

^a ^Chi-square test.

Source: Subject Questionnaire.

### Impact of FM on Productivity

**Figure 3:**
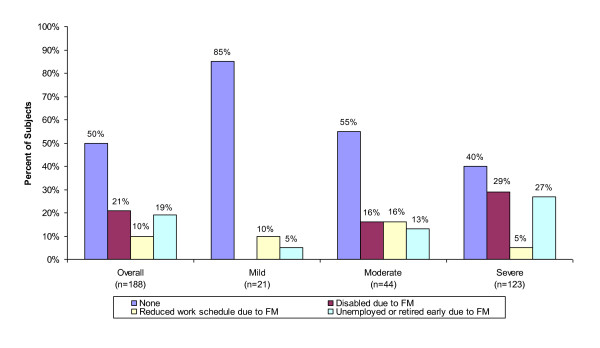
**Impact of Fibromyalgia on Employment: Overall and by Severity Level**. Source: Subject Questionnaire. * P-value < 0.001 (Chi-square test; mild vs. moderate vs. severe by impact of FM on employment status).

Overall, half (50%) of the subjects reported some disruption in their work status due to FM, including reduced work schedule (10%), disabled (21%), unemployed or retired early (19%) (Figure 3). Additionally, 15 percent of mild subjects reported some disruption in their work status due to FM compared to 45 percent of moderate subjects and approximately 60 percent of severe subjects (p < 0.001).

Employed subjects reported missing a mean of 1.8 days of work due to FM over the last 4 weeks (Table 7). Extrapolating, this represents a mean of 23.2 days of work missed per year due to FM. On average, employed subjects reported working with symptoms 15.6 days over the last 4 weeks and being 75 percent effective in their work-related activities when working with symptoms.

**Table 7:** Impact of Fibromyalgia on Productivity over the Past 4 Weeks, Overall and by Fibromyalgia Severity Level

		Severity Level			
	Overall	Mild	Moderate	Severe	p-value^a^
Days missed from work due to FM^b ^					0.030
n	78	14	29	35	
mean (SD)	1.8 (3.9)	0.4 (0.9)	1.0 (1.4)	3.0 (5.4)	
median (range)	0.0 (0.0-28.0)	0.0 (0.0-3.0)	0.0 (0.0-4.0)	1.0 (0.0-28.0)	
Days worked with symptoms related to FM^b^					< 0.001
n	81	15	30	36	
mean (SD)	15.6 (9.0)	6.9 (7.4)	15.3 (7.7)	19.4 (8.2)	
median (range)	17.0 (0.0-28.0)	5.0 (0.0-28.0)	17.0 (0.0-28.0)	20.0 (0.0-28.0)	
Average effectiveness at work on days with FM symptoms^b^					0.002
n	70	11	28	31	
mean (SD)	75.3 (18.3)	89.8 (11.0)	77.7 (15.1)	67.9 (19.7)	
median (range)	80.0 (20.0-100.0)	90.0 (70.0-100.0)	80.0 (30.0-100.0)	75.0 (20.0-100.0)	
Hours of unpaid caregiver help for FM in activities of daily living					0.003
n	203	21	49	133	
mean (SD)	29.1 (88.5)	3.6 (7.0)	22.8 (100.3)	35.4 (90.4)	
median (range)	5.0 (0.0-720.0)	0.0 (0.0 - 30.0)	0.0 (0.0 - 700.0)	8.0 (0.0 - 720.0)	

^a ^Kruskal-Wallis test.

^b ^Among subjects employed for pay (full-time or part-time).

Source: Subject Questionnaire.

The mean number of days missed from work during the past 4 weeks also increased with FM severity with mild subjects reporting missing 0.4 days, moderate reporting 1.0 day, and severe reporting 3.0 days (p = 0.030); corresponding to an annual average of 5, 13, and 39 days of worked missed per year for mild, moderate, and severe FM subjects, respectively (Table 7). Additionally, mild subjects reporting working with symptoms over the past 4 weeks a mean of 6.9 days, moderate reporting 15.3 days, and severe reporting 19.4 days (p < 0.001) This corresponds to an average of 89, 199, and 252 days worked with symptoms annually, for mild, moderate, and severe subjects, respectively. On average, mild, moderate, and severe subjects reported being 90 percent, 78 percent, and 68 percent effective while working with FM symptoms, respectively (p = 0.002).

FM caregivers provided mild, moderate, and severe FM subjects unpaid help for ADLs a mean of 3.6, 22.8, and 35.4 hours over the past 4 weeks, respectively (p = 0.003). This corresponds to an average of 47, 296, and 460 hours a year of unpaid caregiver help for ADLs for mild, moderate, and severe FM subjects, respectively.

## Discussion

This is one of the few studies in the US to comprehensively assess the core FM symptom dimensions identified by OMERACT and to explore the disease burden of FM by disease severity levels. Although the majority of patients in this study were receiving prescription medications to treat FM, they reported high levels of pain, anxiety, depression, sleep disturbance, cognitive dysfunction, and functional limitations. These findings were consistent with some previous study results:

**• Pain, anxiety, and depression - **Over half of all patients reported being in severe pain due to FM, and a majority reported some level of anxiety (70%) or depression (65%), which is consistent with other research [4,7,8].

**• Sleep, shortness of breath and headache - **Overall, patients experienced substantially more sleep problems than the general US population, as indicated by a higher MOS-SS Problem Index score of 57.5, versus the US norm of 25.8 (Figure 2) [26]. There was a difference of 32.6 between the US norm (60.5) and our study (27.9) for the sleep adequacy subscale; indicating that FM patients had less adequate sleep. FM patients in our study also reported higher scores for shortness of breath or headache, sleep disturbance, daytime somnolence, and snoring as compared to the US norms (Figure 2). Our findings are consistent with recent literature reporting that over 90 percent of FM patients suffer from troubled and non-restorative sleep [27].

**• Cognitive dysfunction - **A majority of our study sample reported cognitive dysfunction with over 88 percent of patients reporting at least some limitations in concentration, making decisions, thinking, and memory due to FM. This is consistent with a case-control study which showed that FM patients performed significantly worse on several measures of cognitive function (working memory capacity, free recall, recognition memory and verbal knowledge) than age-matched controls and that patients' performance on these measures were similar or worse than adults 20 years older [6].

**• Functional limitations **- Our findings also are consistent with published studies showing FM patients report much higher functional limitations compared to the general population. In our study, the mean FIQ score was 63.2. A Canadian study that compared FM patients with general population controls found a mean FIQ score of 61.2 among FM patients versus 21.9 among the controls [5]. A national survey of women in the US with FM and a mean age of 47 years, found that the mean level of physical functioning in their overall sample was lower than that for an average 80 to 89 year old [3].

Patients also demonstrated diminished global health and HRQoL. Mean EQ-5D scores for subjects in our study, overall (0.35) and at all FM severity levels (0.76 mild; 0.57 moderate; 0.20 severe), were substantially lower than the mean EQ-5D score for the US population age 45 to 54 (0.87) [28]. These results are consistent with other studies reporting substantial health status limitations among FM patients [29,30] and that FM patients have worse SF-36 scores on the subscales of physical functioning, role functioning (emotional and physical), body pain, general health, vitality, social functioning, and mental health compared with the general population [7,30].

Perhaps due to the large pain burden and detrimental impact on various domains of health, the overall HRQoL found in FM patients, especially in those with severe form of disease, was not only worse than that of the general population but also in comparison with many other chronic conditions reported in the utility and generic HRQoL literature [7]. This finding also was in line with studies comparing FM with similar conditions. In particular, FM patients were shown to have worse HRQoL than patients with rheumatoid arthritis, and patients with non-inflammatory rheumatic disorders and systemic lupus erythematosus [14,31].

Our study showed that FM has a substantial negative impact on productivity, with the overall sample reporting an average of more than 23 days missed from work per year. A meta-analysis of FM burden reviewed studies that reported that FM patients missed between 11 and 31 days of work per year [7]. Of those who were unemployed in our study, 38 percent reported they were disabled due to FM. This is consistent with literature reporting 31 percent of FM patients in a sample were disabled due to FM [5].

This is the first US study to explore the impact of FM on caregiver productivity. The study found that, on average, caregivers spent the equivalent of almost 10 work weeks (378 hours) providing unpaid help to patients with FM per year. This finding highlights the importance of incorporating caregivers' time when assessing the full economic impact of FM on society.

This study is also the first in the US to comprehensively report the burden of FM by disease severity levels. FM severity was negatively and significantly associated with most patient-reported outcomes, such as pain intensity, anxiety and depression, sleep problems, cognitive dysfunction, as well as overall HRQoL and utility scores. Severe FM also was more likely to be accompanied by comorbid depression, anxiety, or chronic fatigue syndrome than mild or moderate FM.

This new understanding of FM by severity level is very important for the evaluation of FM treatments and priority setting in health care, for example by providing input data for the evaluation of medical resource utilization, costs and quality of life adjustments in the health economic evaluation of alternative FM treatments.

Although this study was comprehensive, it had some possible limitations. Only practices that volunteered to participate were included in the study. Although there is no specific reason to assume that these sites may differ from other practices that treat FM, there may be unknown underlying variations. Additionally, data were collected from patients who were actively seeking care; we do not have similar objective data for patients who did not enroll. Therefore, our results may not be generalizable to the overall FM population. Our study's inclusion criteria required that patients have experienced pain in the past 24 hours; perhaps biasing our overall sample towards more severe FM. Therefore, results for the overall FM population should be taken with some caution, and where appropriate, results reported by severity groups should be used as comparative or input data in future research. Finally, this study was cross-sectional, and therefore we cannot establish causality, only the association between FM and outcomes.

## Conclusion

This study represents one of the first attempts to characterize the full patient experience of FM patients' disease, function, HRQoL, and productivity losses in the US. Although the majority of patients were receiving medical attention and prescription medications for FM, they reported high levels of pain, anxiety, depression, sleep disturbance, cognitive dysfunction, and diminished HRQoL, as well as substantial losses in productivity for them and their caregivers. Patients' health status and other key symptom domains worsened as FM severity increased. These results highlight the disease burden and limitations of treatment options currently available in the US.

## List of Abbreviations

ADLs: Activities of Daily Living; CRF: Case Report Form; EQ-5D: EuroQol 5 Dimensions; FIQ: Fibromyalgia Impact Questionnaire; FM: Fibromyalgia; GFI: Global Fatigue Index; HADS: Hospital Anxiety and Depression Scale; HRQoL: Health Related Quality of Life; MAF: Multidimensional Assessment of Fatigue; MOS-SS: Medical Outcomes Study - Sleep Scale; MTPS: Manual Tender Point Survey; OMERACT: Outcome Measures in Rheumatology Clinical Trials; SD: Standard Deviation; US: United States.

## Competing interests

This study was funded by Pfizer, Inc. Arthi Chandran and Gergana Zlateva are employees of Pfizer Inc. Caroline Schaefer, Meghan Hufstader, and Rebecca Baik are employees of Covance Market Access Services Inc, and served as paid consultants to Pfizer during the conduct of this study and the development of this manuscript.

## Authors' contributions

CS, MH, RB, AC and GZ were integral to this study and to the development of this manuscript MM, DG, and RG served as clinical reviewers and contributors to the analysis and discussion sections. All authors have read and approved the final manuscript.
